# Optimizing Large-Scale COVID-19 Nucleic Acid Testing with a Dynamic Testing Site Deployment Strategy

**DOI:** 10.3390/healthcare11030393

**Published:** 2023-01-30

**Authors:** Xiaozhou He, Li Luo, Xuefeng Tang, Qingyi Wang

**Affiliations:** 1 Business School, Sichuan University, Chengdu 610065, China; 2 Management Science and Operations Research Institute, Sichuan University, Chengdu 610065, China; 3 Sichuan Center for Disease Control and Prevention, Chengdu 610041, China

**Keywords:** COVID-19 control, large-scale testing, dynamic site deployment, spatial–temporal demands

## Abstract

The COVID-19 epidemic has spread worldwide, infected more than 0.6 billion people, and led to about 6 million deaths. Conducting large-scale COVID-19 nucleic acid testing is an effective measure to cut off the transmission chain of the COVID-19 epidemic, but it calls for deploying numerous nucleic acid testing sites effectively. In this study, we aim to optimize the large-scale nucleic acid testing with a dynamic testing site deployment strategy, and we propose a multiperiod location-allocation model, which explicitly considers the spatial–temporal distribution of the testing population and the time-varied availability of various testing resources. Several comparison models, which implement static site deployment strategies, are also developed to show the benefits of our proposed model. The effectiveness and benefits of our model are verified with a real-world case study on the Chenghua district of Chengdu, China, which indicates that the optimal total cost of the dynamic site deployment strategy can be 15% less than that of a real plan implemented in practice and about 2% less than those of the other comparison strategies. Moreover, we conduct sensitivity analysis to obtain managerial insights and suggestions for better testing site deployment in field practices. This study highlights the importance of dynamically deploying testing sites based on the target population’s spatial–temporal distribution, which can help reduce the testing cost and increase the robustness of producing feasible plans with limited medical resources.

## 1. Introduction

The coronavirus disease of 2019 (COVID-19) epidemic has widely spread worldwide and led to more than 0.66 billion confirmed patients and about 6.7 million deaths up to September 2022 [1]. To control the epidemic, various policies and measures have been implemented, such as implementing a lockdown policy [2], keeping social distance to reduce the infection rate [3], developing various COVID-19 vaccines to help protect the uninfected people [4], isolating the suspected or confirmed individuals [5], treating infected patients in dedicated emergency hospitals [6], and conducting large-scale COVID-19 nucleic acid testing (NAT) to screen the infected individuals [7]. In the early stage of the COVID-19 epidemic, many countries implemented the lockdown policy, which helped control the severe outbreak of the epidemic at the high price of socioeconomic damage. For example, in India, about 1.3 billion people were severely influenced by the lockdown, and many of them lost jobs [8], and the U.S. lost about USD 65.3 billion a month during lockdown [9]. To unlock people and recover social activity, it is important to quickly identify and cut off the transmission chain of the COVID-19 epidemic, especially in densely populated cities, and this requires conducting large-scale COVID-19 NATs effectively in post-lockdown years [10].

Severe epidemics can be effectively controlled with the help of large-scale NATs [11] since large-scale NATs not only allow earlier identification of asymptomatic and presymptomatic carriers [12] but also help dynamically monitor the prevalence of the virus in the population across various areas [13]. Many countries have implemented large-scale COVID-19 NATs. For example, a repeated nationwide NAT was conducted in Slovakia in November 2020 [14]; Luxembourg conducted an NAT to cover everyone in May 2020 [15]; a citywide NAT was launched in Liverpool, U.K. [16]. More recently, Chengdu, a megacity located in southwestern China, implemented a large-scale NAT, which tests 20 million residents each day, to completely stop the spread of COVID-19 in two weeks. In practice, large-scale COVID-19 NATs can be conducted in two ways, i.e., at-home tests and centralized tests. Compared with the at-home tests, centralized tests can benefit from a group testing strategy (i.e., combining samples from multiple individuals into a single pool to test) and enhance the effectiveness and efficiency of large-scale COVID-19 NATs [17,18]. However, even with the group testing strategy, the costs of large-scale COVID-19 NATs can be still very high due to the overwhelming testing demands. For example, China finished 11.5 billion nucleic acid tests till April 2022 [19], and the U.S. conducted 2 million NATs each week till May 2020 [20]. Considering that new epidemics or new waves of the COVID-19 epidemic may appear in the near future and that the cost associated with large-scale NATs can be very high, planning and implementing large-scale NATs effectively and efficiently is vital to reducing the large-scale NAT cost and improving the preparedness and responses to potential large-scale epidemics in the future.

**Figure 1:**
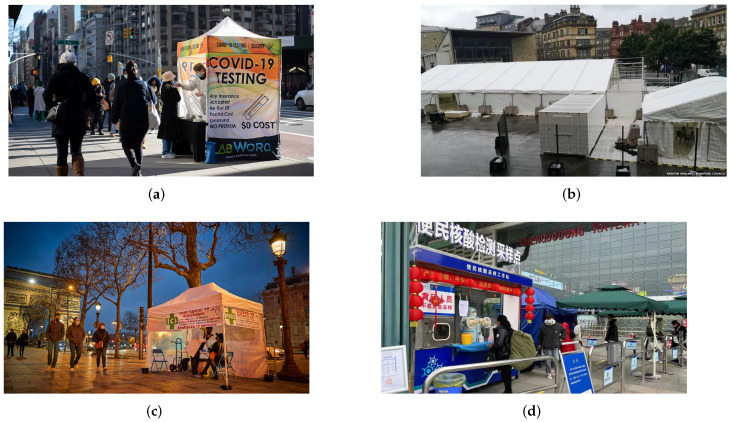
Illustration of the various COVID-19 NAT sites deployed worldwide. (**a**) An NAT site in New York City, U.S. [27]; (**b**) An NAT site in Bradford, U.K. [28]; (**c**) An NAT site in Paris, France [29]; (**d**) An NAT site in Chengdu, China.

The effectiveness and efficiency of large-scale NATs can be improved in several ways, such as optimizing the supply network for NAT kits [21], optimizing the service area of NAT facilities [22], and optimizing the deployment of NAT sites [23], which is the focus of this study. In practice, various types of NAT sites, as shown in Figure 1, are deployed worldwide to serve the target populations who need testing. Specifically, in the U.S., some community-based testing sites and point-of-care facilities are deployed at some pharmacies, health centers, physician offices, urgent care facilities, temporary locations, etc., to conduct large-scale COVID-19 NATs [24,25]. In the U.K., the NAT sites are mainly deployed at clinics, pharmacies, and healthcare centers [26]. In China, the NAT sites are mainly deployed at hospitals, clinics, pharmacies, and other temporary sites along the main streets.

The numbers and locations of the deployed NAT sites can influence the accessibility and expenditure of NATs significantly, and various types of medical resources, e.g., staff and medical supplies, are needed to run a deployed NAT site. Specifically, NAT accessibility can be improved by setting up 15 min NAT circles with a reasonable budget [30]. Our field survey indicates that a deployed NAT site requires medical supplies such as protective suits, marks, and swabs, and is generally operated by several testing units (each testing unit has 3–5 staff to test, distribute the testing cotton swabs, record the personal information, and keep order and security). If few people are expected to show up at a testing site in a period, that site can be temporarily closed to reduce the cost of NATs without sacrificing service accessibility. However, in most practices, the NAT sites are statically deployed in all periods, which can lead to serious supply–demand imbalance (testing capacity overflow or waste) problems in some periods. To tackle these practical problems, a dynamic testing site deployment strategy should be implemented to generate more flexible NAT site deployment plans.

This study aims to optimize the large-scale nucleic acid testing with a dynamic testing site deployment strategy, and we propose a multiperiod location-allocation model, which explicitly considers the spatial–temporal distribution of the testing population and the time-varied availability of various testing resources. Our main contributions are threefold: first, we develop a multiperiod location-allocation model following a dynamic site deployment strategy to facilitate large-scale NATs considering the limitation of multitype medical resources and the movement of demands to the nearest deployed NATs; second, we investigate various static NAT site deployment strategies to show outperformance of the dynamic strategy and to generate theoretical insights; third, we conduct a real-world case study on Chengdu, China, to verify the effectiveness of our proposed model and to obtain managerial insights for better field practice of large-scale NATs.

The rest of the article is organized into four sections. Section 2 reviews relevant studies. Section 3 formally describes the problem and presents our multiperiod location-allocation model. Section 4 conducts a case study to obtain managerial insights. Section 5 draws the conclusions and discusses future work briefly.

## 2. Literature Review

Our literature review mainly covers location planning studies on non-emergency and emergency healthcare facilities (EHFs). While non-emergency healthcare facility planning studies pave a solid foundation for our study, the studies on EHFs, which are more closely related to ours since NAT sites are an example of EHFs, help point out the research gap.

In the literature, the location planning problems of various healthcare facilities such as community hospitals, medical healthcare centers, and drugstores are widely studied [31]. In the early studies, the regional healthcare demands are assumed to be deterministic, and classic location planning models, such as the *p*-median model, maximum covering model, and set covering model, are employed to optimize the locations of long-term care facilities [32], perinatal facilities [33], community health centers [34], and blood banks [35]. Moreover, multiperiod situations are considered in the location planning of healthcare facilities for nomadic dwellers who experience a seasonal movement [36]. Uncertainties related to the healthcare facility locations, such as the stochastic demands for hospitals [37] and the uncertain demands and transportation cost in a medical services network [38,39], are also addressed.

As the occurrence of various disasters, such as earthquakes, floods, hurricanes, mass-casualty incidences, and epidemics, becomes increasingly more frequent in the past two decades, the location planning problems related to the deployment of EHFs attract lots of research efforts. In general, the EHFs can be categorized as permanent and temporary facilities. The deployment of permanent EHFs, such as ambulance stations [40,41], emergency centers [42], and trauma centers [43], are optimized with the goals of covering demands effectively and providing emergency medical services efficiently. Specifically, Cho et al. [44] optimized the locations of trauma centers, associated helicopter platforms, and helicopter depots simultaneously. Chan et al. [45] investigated the problem of deploying the public automated external defibrillators and developed a general optimization framework for three situations. Based on real data from an emergency medical service system, Nasrollahzadeh et al. [46] built a model to develop high-quality solutions for real-time ambulance dispatching and relocation management. Besides the permanent EHFs deployed for general emergency medical service, some other permanent EHFs are deployed before various disasters considering uncertain disaster impacts. For example, Mete and Zabinsky [47] proposed a two-stage stochastic programming model to optimize the location and inventory level of medical supply before disasters and the supply distribution after disasters. Jenkins et al. [48] proposed a robust location-allocation model to tackle a medical evacuation problem, which determines the locations of mobile aeromedical staging facilities and allocations of aeromedical helicopters for military operations.

Temporary emergency healthcare facilities are significant to increase the limited capacity of emergency medical services and serve victims better during and after disasters [49]. The existing studies investigate the deployment of various temporary EHFs, including temporary emergency medical centers [50,51], alternative care sites [52], points of dispensing [53], and NAT sites [23]. Specifically, Chen and Yu [54] optimized the locations of temporary emergency medical service facilities considering the existing hospitals and transportation infrastructure in post-disaster responses. Sharma et al. [55] proposed a location-allocation model for dynamically deploying temporary blood banks during and after disasters. Tang et al. [56] developed a multiperiod vaccination planning model to optimize the opening and closing of vaccine sites in various periods. Luo et al. [57] built a multiperiod location-allocation model for deploying emergency healthcare facilities and managing various types of patients integrally during COVID-19 epidemics. Some other studies focus on the deployment of COVID-19 NAT laboratories. Devi et al. [58] proposed a location-allocation model to deploy temporary testing laboratories for surging susceptible and infected individuals in India. Hosseini-Motlagh et al. [21] further considered the location planning of mobile testing labs in developing a supply network for COVID-19 NAT kits. On the contrary, to the best of our knowledge, the studies on the deployment or locations of NAT sites are rather limited. Fan and Xie [22] addressed a territory design problem, which optimizes the service area of each NAT facility and considers purchasing insufficient resources from third-party medical institutions at high prices. Risanger et al. [59] optimized the selection of pharmacies for COVID-19 testing with a goal of maximizing the size of the population who travel to their nearest selected pharmacy. Villicana-Cervantes and Ibarra-Rojas [60] planned locations of COVID-19 testing labs considering several facility accessibility indicators and the service areas of labs. Both [59] and [60] were based on the *p*-center location model, and they limited the maximum number of testing facilities and ignored constraints related to the service capacity, medical supplies, and testing staff and the fixed cost of facility deployment. In particular, Liu et al. [23] focused on optimizing the locations and the time-varied supply capacities of NAT facilities, which are similar to test kit warehouses and delivery test kits to the demand points in each period. Although the studies of [23] and ours both focus on the dynamic management of NAT facilities, the facilities themselves are different, and our NAT sites can be viewed as the demand points in [23].

In sum, our literature review points out two research gaps. First, although many previous works contribute to controlling the COVID-19 epidemic, few studies focus on enhancing the NAT operation, especially via optimizing the NAT site deployment. Second, the travel cost associated with the target population, who has a dynamically changed spatial–temporal distribution and normally self-move to their nearest emergency medical facility in each period, is seldom considered. To fill the gaps, we tackle the NAT site deployment problem, which explicitly considers the time-varied spatial–temporal distribution of the target population and the time-varied availability of various testing resources in this study.

## 3. Problem Statement and Model Formulation

**Figure 2:**
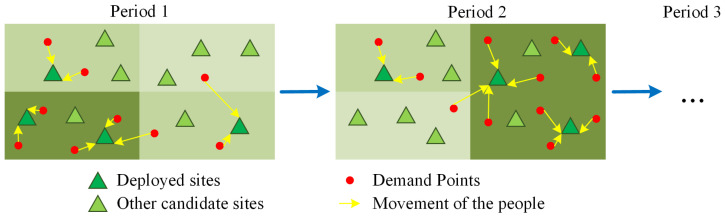
Illustration of the planning problem.

We illustrate our dynamic site deployment problem with Figure 2. As illustrated by the green squares with different shades (the deeper shade, the more population), the spatial distributions of the population in a city are time-varying, which can be due to that on weekdays, people attend work downtown in the morning and return home at residential areas at night. Due to the time-varied population distribution, the COVID-19 NAT demands (denoted with red circles) also vary in time and space, and this motivates a dynamic site deployment strategy, which dynamically opens and closes the candidate COVID-19 NAT sites, to be implemented for better supply–demand balance. Based on field practices, we assume that the target people self-move to their nearest deployed testing sites (shown by the yellow arrows) in each period, and we aim to optimize the dynamic NAT sites deployment plan under a cost-minimization goal.

We consider a planning horizon, which includes τ periods, and we contain all periods in a set *T*, i.e., T={1,2...,τ}. We let all demand points and all candidate NAT sites be contained in sets *I* and *J*, respectively. Moreover, various types of medical supplies and staff, which are important for the COVID-19 NAT, form sets *K* and *H*, respectively. Due to the movement of the target population, the demand amount varies in time and space, and we let $D_{it}$ be the demand amount at demand point i∈I in period t∈T. We denote the distance between each demand point i∈I to each candidate NAT site j∈J as $l_{ij}$. Moreover, for each candidate NAT site j∈J, we denote its testing capacity (the maximum amount of demand that can be served) per period, deployment cost, and unit penalty costs of capacity waste (overflow) as $C_{j}$, $f_{j}$, and $\alpha_{j}$ ($\beta_{j}$), respectively. To run an NAT site j∈J for one period, $a_{kj}$ amount of types k∈K medical supplies and $b_{hj}$ number of type h∈H staff are required. As the emergency medical resources for large-scale COVID-19 NAT are relatively limited and time-varied, we denote the total amount (number) of type k∈K medical supplies (type h∈H staff) available in period t∈T as $A_{kt}$ ($B_{ht}$). Finally, to measure the accessibility, we let δ be a factor transferring people’s walking distance into cost, and denote *M* as a huge positive number.

The key decisions of dynamically opening NAT sites are denoted with binary decision variables, $y_{jt}\in \left\{0,1\right\}$, which is 1 if candidate NAT site j∈J opens in period t∈T, and is 0 otherwise. Since we assume that the target population of each demand point will self-move to the nearest opened NAT site, the site location decisions ($y_{jt}$) will also determine the spatial allocation of demands to the opened sites in each period. Thus, we define auxiliary variables $x_{ijt}\geq 0$ to evaluate the number of target people of demand point i∈I that self-move to candidate NAT site j∈J in period t∈T. Moreover, due to the spatial–temporal variation of demands, the capacity of opened NAT sites can be left or exceeded. Thus, we employ $c_{jt}^{-}\geq 0$ and $c_{jt}^{+}\geq 0$ to evaluate the amount of left capacity and the amount of exceeded capacity at candidate NAT site j∈J in period t∈T.

The notations of sets, parameters, and decision variables are summarized in Table 1.

With the above notations, the multiperiod location-allocation Model (P) is formulated as follows:

(1) $$\begin{array}{cc} \left(P\right)\;\min\; & \sum_{t\in T}\sum_{j\in J}f_{j}y_{jt}+\sum_{t\in T}\sum_{j\in J}\left(\alpha_{j}c_{jt}^{-}+\beta_{j}c_{jt}^{+}\right)+\delta \sum_{t\in T}\sum_{i\in I}\sum_{j\in J}l_{ij}x_{ijt}, \end{array}$$

(2) $$\begin{array}{cc} s.t.\; & \sum_{j\in J}x_{ijt}=D_{it},\;\forall i\in I,t\in T, \end{array}$$

(3) $$\begin{array}{cc} & x_{ijt}\leq My_{jt},\;\forall i\in I,j\in J,t\in T, \end{array}$$

(4) $$\begin{array}{cc} & x_{ijt}\leq \left(1-y_{j^{\prime }t}\right)M,\;\forall i\in I,j\in J,j^{\prime }\in J:l_{ij^{\prime }}<l_{ij},t\in T, \end{array}$$

(5) $$\begin{array}{cc} & \sum_{j\in J}a_{kj}y_{jt}\leq A_{kt},\;\forall k\in K,t\in T, \end{array}$$

(6) $$\begin{array}{cc} & \sum_{j\in J}b_{hj}y_{jt}\leq B_{ht},\;\forall h\in H,t\in T, \end{array}$$

(7) $$\begin{array}{cc} & c_{jt-1}^{+}+\sum_{i\in I}x_{ijt}=c_{jt}^{+}+C_{j}y_{jt}-c_{jt}^{-},\;\forall j\in J,t\in T:t\neq 1, \end{array}$$

(8) $$\begin{array}{cc} & \sum_{i\in I}x_{ijt}=c_{jt}^{+}+C_{j}y_{jt}-c_{jt}^{-},\;\forall j\in J,t=1, \end{array}$$

(9) $$\begin{array}{cc} & y_{jt}M\geq c_{jt-1}^{+},\;\forall j\in J,t\in T:t\neq 1, \end{array}$$

(10) $$\begin{array}{cc} & y_{jt}\in \left\{0,1\right\},\;\forall j\in J,t\in T, \end{array}$$

(11) $$\begin{array}{cc} & x_{ijt}\geq 0,\;\forall i\in I,j\in J,t\in T, \end{array}$$

(12) $$\begin{array}{cc} & c_{jt}^{-}\geq 0,\;\forall j\in J,t\in T, \end{array}$$

(13) $$\begin{array}{cc} & c_{jt}^{+}\geq 0,\;\forall j\in J,t\in T. \end{array}$$

The objective function (1) minimizes the total cost of COVID-19 NAT for all periods, which includes the NAT site deployment cost (the first term), the penalty costs related to the testing capacity waste (the second term) and overflow (the third term), and the weighted travel cost of all target populations (the last term). Specifically, the NAT site deployment cost is incurred by setting up the facility, e.g., preparing testing supplies and dispatching staff. The testing capacity waste and overflow penalty costs are due to the imbalance between the dynamic testing demand and the prepared testing capacity. When the testing demand is lower (higher) than the prepared testing capacity, a capacity waste (overflow) penalty is caused. The weighted travel cost evaluates the NAT service accessibility of the target population, and we assume that the travel cost is proportional to the distance.

Constraints () ensure that in each period *t*, all target populations of each demand point *i* will self-move to an NAT site *j*. Constraints () and () together enforce that the target populations of each demand point will self-move to their nearest opened COVID-19 NAT site in each period. Constraints () and () limit the maximum amount of supply and the maximum number of staff available for deploying NAT sites in each period, respectively. Constraints () and () are flow balance constraints at each candidate NAT site for periods 2 to τ and period 1, respectively. Constraints () ensure that if there is an overflow at an NAT site in period *t*, then that site must be deployed in period t+1 as well. Constraints ()–() set bounds for the binary and non-negative continuous decision variables.

To show the benefits of the dynamic site deployment strategy, we compare the dynamic site deployment strategy with the real deployment plan implemented in practices and other static strategies, which keep the site deployment unchanged for all periods. We denote the dynamic site deployment strategy as the base strategy (BS) and let the real plan be Comparison Strategy I (CS-I). We denote a static strategy, which obtains a static site deployment plan based on the time-averaged demands of all periods, as Comparison Strategy II (CS-II), and CS-II leads to the following static (single-period) Model (SP):

(14) $$\begin{array}{cc} \left(\mathrm{SP}\right)\;\min\; & \sum_{j\in J}f_{j}{\hat{y}}_{j}+\sum_{j\in J}\left(\alpha_{j}{\hat{c}}_{j}^{-}+\beta_{j}{\hat{c}}_{j}^{+}\right)+\delta \sum_{i\in I}\sum_{j\in J}l_{ij}{\hat{x}}_{ij}, \end{array}$$

(15) $$\begin{array}{cc} s.t.\; & \sum_{j\in J}{\hat{x}}_{ij}={\bar{D}}_{i},\;\forall i\in I, \end{array}$$

(16) $$\begin{array}{cc} & {\hat{x}}_{ij}\leq M{\hat{y}}_{j},\;\forall i\in I,j\in J, \end{array}$$

(17) $$\begin{array}{cc} & {\hat{x}}_{ij}\leq \left(1-{\hat{y}}_{j^{\prime }}\right)M,\;\forall i\in I,j\in J,j^{\prime }\in J:l_{ij^{\prime }}<l_{ij}, \end{array}$$

(18) $$\begin{array}{cc} & \sum_{j\in J}a_{kj}{\hat{y}}_{j}\leq {\bar{A}}_{k},\;\forall k\in K, \end{array}$$

(19) $$\begin{array}{cc} & \sum_{j\in J}b_{hj}{\hat{y}}_{j}\leq {\bar{B}}_{h},\;\forall h\in H, \end{array}$$

(20) $$\begin{array}{cc} & \sum_{i\in I}{\hat{x}}_{ij}={\hat{c}}_{j}^{+}+C_{j}{\hat{y}}_{j}-{\hat{c}}_{j}^{-},\;\forall j\in J, \end{array}$$

(21) $$\begin{array}{cc} & {\hat{y}}_{j}\in \left\{0,1\right\},\;\forall j\in J, \end{array}$$

(22) $$\begin{array}{cc} & {\hat{x}}_{ij}\geq 0,\;\forall i\in I,j\in J, \end{array}$$

(23) $$\begin{array}{cc} & {\hat{c}}_{j}^{-}\geq 0,\;\forall j\in J, \end{array}$$

(24) $$\begin{array}{cc} & {\hat{c}}_{j}^{+}\geq 0,\;\forall j\in J, \end{array}$$

where ${\bar{D}}_{i}=\frac{\sum_{t\in T}D_{it}}{\tau },\forall i\in I$, ${\bar{A}}_{k}=\frac{\sum_{t\in T}A_{kt}}{\tau },\forall k\in K$, and ${\bar{B}}_{h}=\frac{\sum_{t\in T}B_{ht}}{\tau },\forall h\in H$, are the time-averaged values of $D_{it}$, $A_{kt}$ and $B_{ht}$, respectively, and the static version of the dynamic decision variables $y_{jt}$, $x_{ijt}$, $c_{jt}^{+}$, and $c_{jt}^{-}$ are denoted with a hat, correspondingly. Moreover, we consider Comparison Strategy III (CS-III), which produces a static deployment plan by ensuring that the site deployment plan is unchanged in each period, and it is produced by Model (TP), which is equivalent to adding extra constraints

(25) $$y_{jt+1}=y_{jt},\forall j\in J,t\in T:t\neq \tau$$

to Model (P).

There exist some properties for the above three strategies BS, CS-II, and CS-III, and the corresponding Models (P), (SP), and (TP).

**Lemma** **1.** *With Constraint (25), Constraints (5) and (6) are tighter than Constraints (18) and (19), respectively.*

**Proof.**  With Constraint (25), the resource constraints of (TP) are equivalent to $\sum_{j\in J}a_{kj}{\hat{y}}_{j}\leq \min_{t\in T}A_{kt},\;\forall k\in K$ and $\sum_{j\in J}b_{hj}{\hat{y}}_{j}\leq \min_{t\in T}B_{ht},\;\forall h\in H$. As $\min_{t\in T}A_{kt}\leq {\bar{A}}_{k}$ and $\min_{t\in T}B_{ht}\leq {\bar{B}}_{h}$, the lemma is thus proved. □

**Proposition** **1.** *(a) Any feasible solution of Model (TP) is feasible to Model (P).*

*(b) Any feasible solution of Model (TP) can induce a feasible solution to Model (SP).*

*(c) A feasible solution of Model (SP) may not be a feasible solution to Model (P) with Constraint (25) added.*

**Proof.**  (a) is obvious and (c) is the direct corollary of Lemma 1. We only prove (b). For (b), we declare that if ${\left(y_{t}^{*},x_{t}^{*}\right)}_{t\in T}$ is a feasible solution of Model (TP), then there exists a solution $\left({\hat{y}}^{*},{\hat{x}}^{*}\right)$ feasible to Model (SP), where ${\hat{y}}^{*}=y_{t}^{*},\forall t\in T$. With Lemma 1 we know that ${\hat{y}}^{*}$ is feasible to Constraints () and (), and with given values of $\hat{y}$ there is a unique group of values of $\hat{x}$ to other constraints of Model (SP) since the values of ${\hat{c}}^{+}$ and ${\hat{c}}^{-}$ are flexible and *M* is big enough for ${\hat{x}}^{*}$ to satisfy demands $\bar{D}$. Consequently, (b) is proved. □

Proposition 1 indicates that the plan given by CS-II may not fit for the real situation as BS defines, except that the resources are abundant, whereas CS-III always produces a feasible plan for the real situation. This proposition also implies that the decision-maker can obtain feasible plans with CS-II and CS-III, as it takes much less time to solve Models (SP) and (TP) than to solve Model (P), especially for large-scale problem instances.

## 4. Case Study

In this section, we present a case study on the COVID-19 NAT site deployment in Chengdu, China. Although Chengdu has some top hospitals, such as the West China Hospital (ranked second in China), and many high-quality healthcare resources, the amount of healthcare resources is far from enough for Chengdu to conduct large-scale COVID-19 NAT in a short time, which makes it important to deploy the NAT sites dynamically and to utilize the limited NAT resources sufficiently. In August 2022, a large-scale COVID-19 NAT is implemented in the Chenghua District of Chengdu to prevent the spread of COVID-19 all over Chengdu city. Chenghua District locates in the northeastern urban area of Chengdu, covers an area of 109.3 square kilometers, and has a resident population of about 1.4 million, which makes the large-scale COVID-19 NAT in Chenghua overwhelming. To finish the COVID-19 NAT mission effectively, the government statically deployed about 110 COVID-19 NAT sites all over the Chenghua district [61], and lots of emergency medical supplies and healthcare staff are dynamically mobilized from other districts to Chenghua district. However, the field practice witnesses that lots of the deployed NAT sites and the limited supplies and staff are wasted due to that in some periods, fewer residents move to some deployed sites to take testing. In the following, we first introduce the parameter settings based on the real-world case of Chenghua District, then present the optimal results of various strategies, and finally conduct sensitivity analysis on some key parameters to obtain managerial insights.

### 4.1. Parameter Settings

**Figure 3:**
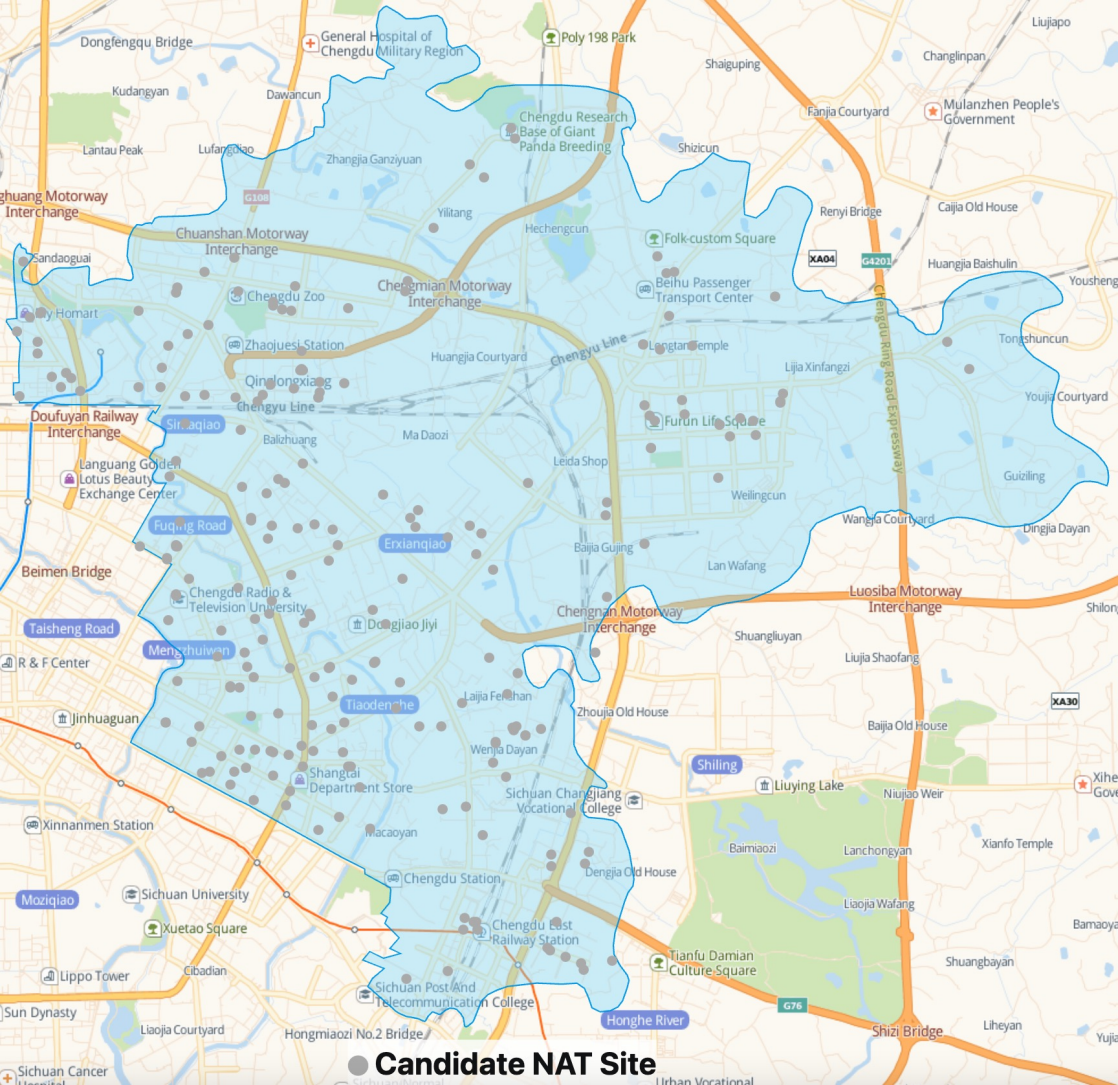
All candidate NAT sites.

In this case study, we consider 686 (|I|=686) demand points, which are distributed all over Chenghua District. Based on the limited real data collected from open channels and our field survey, a total of 232 (|J|=232) candidate NAT sites are incorporated, and the locations of the candidate sites are illustrated in Figure 3. We consider a planning horizon of four periods (T={1,2,3,4} and τ = 4) on a weekday, and periods 1–4 stand for 6:00–10:00, 10:00–14:00, 14:00–18:00, and 18:00–22:00, respectively. For simplicity, two types of medical supplies (|K|=2), i.e., testing supplies (k=1) and PPEs (k=2), and two kinds of staff (|H|=2), i.e., testing staff (h=1) and other staff (h=2), are considered in this case.

**Figure 4:**
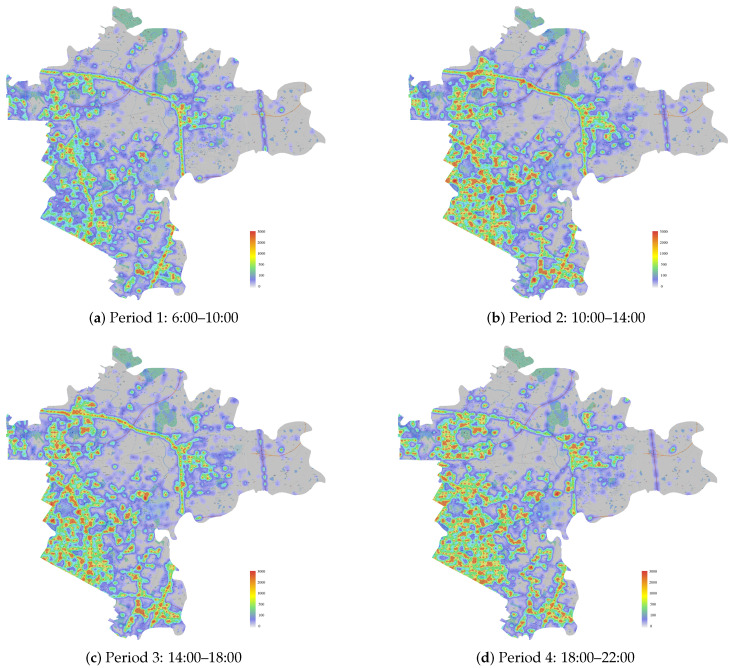
Thermodynamic diagram for real spatial–temporal population data in Chenghua District.

The time-varied demand amounts of each demand point $D_{it}$ are estimated based on the real spatial–temporal population data, which are illustrated in the thermodynamic diagram in Figure 4 and is obtained from the population dynamic thermodynamic diagram of Baidu Maps [62]. Specifically, we assume that the demands of periods 1–4 are about 0.5%, 1.5%, 3%, and 1% of the corresponding period’s population number in Figure 4.

The distances between each demand point i∈I and each candidate NAT site j∈J, $l_{ij}$, are further produced via employing the application programming interface of Gaode Maps [63]. Considering the convenience and accessibility requirements of COVID-19 NAT, we assume that the target people have a maximum moving distance of 3 kilometers, which helps remove the distances greater than 3km to finally generate 22,771 valid distance values.

According to reality, we set the parameters related to the 232 candidate NAT sites by classifying all candidate NAT sites into five types, including 57 general hospitals (Type I), 68 clinics (Type II), 41 healthcare centers (HCs, Type III), 44 pharmacies (Type IV), and 22 other candidate NAT sites (Type V), respectively. We list the parameter settings for the five types of candidate NAT sites in Table 2. While $C_{j}$ and $f_{j}$ are set according to field practice reports and news, the other parameters ($\alpha_{j}$, $\beta_{j}$, $a_{kj}$ and $b_{hj}$) are produced via educated guessing. For example, a testing unit can test 100–120 people per hour, and, thus, the testing capacity is around 400 per period (four hours) for each clinic or pharmacy, which normally has only one testing unit.

The total amounts of available supplies $A_{kt}$ and the total number of available staff $B_{ht}$ in each period, set according to field investigations and reasonable guessing, are listed in Table 3. Finally, we let δ, the factor transferring distance into cost, be 0.1 in the base case. We believe that a decision-maker can set the values of the various parameters of Model (P) better according to more realistic data and information in practice. Our case leads to a planning instance of about 2,271,628 constraints and 93,868 variables (928 binary variables).

We solve (P) and the models associated with the CS-II and CS-III with the state-of-the-art mixed-integer programming solver Gurobi, and we evaluate the performance of the real plan, CS-I, by fixing its static site deployment plan in (P) and solving (P) again.

### 4.2. Optimal Results

The optimal objective values of the various strategies are compared in Table 4. When the base case parameter settings are applied, CS-I is infeasible since some demand points cannot be served by any deployed site due to the 3km maximum serving distance assumption. This indicates that dynamically deploying NAT sites provided by BS can contribute to producing feasible plans, even considering relatively short serving distances. To ensure that all strategies are optimally solved, we set the maximum serving distance as 3.5 km for CS-I. We find that BS, via implementing the dynamic NAT site deployment strategy, obtains an optimal total cost that is better than those of the other static comparison strategies. Specifically, the optimal total cost is about 15%, 2%, and 2% lower than those of CS-I, CS-II, and CS-III, respectively. We find that all cost components of CS-I are higher than those of BS, which highlights that our model can help to improve field practice greatly and benefit both the government and the target population. In particular, the deployment (capacity waste) cost of BS is about 65% (50%) of that of CS-I, which shows the benefits of BS in reducing the number (capacity waste) of deployed NAT sites. Although compared with CS-II and CS-III, the optimal total cost of BS is just about 2% (0.086 million) less, which seems to be marginal for Chenghua District, it can help to reduce cost significantly (in millions) considering that the COVID-19 NAT can be conducted at a greater scale, implemented at more districts or cities and covering much more target population for more periods. Moreover, we find that while the optimal total costs of BS, CS-II, and CS-III are similar, it takes less (the least) time to solve the model of CS-III (CS-II) optimally. This suggests that when the testing supplies and staff are abundant (insufficient) in each period and the planning time is limited, CS-II (CS-III) can be employed to find a good substitute for the optimal site deployment plan quickly. In short, Table 4 not only shows the benefits and effectiveness of the dynamic site deployment strategy implemented by (P) but also suggests promising static strategies for field practices under some special situations.

**Figure 5:**
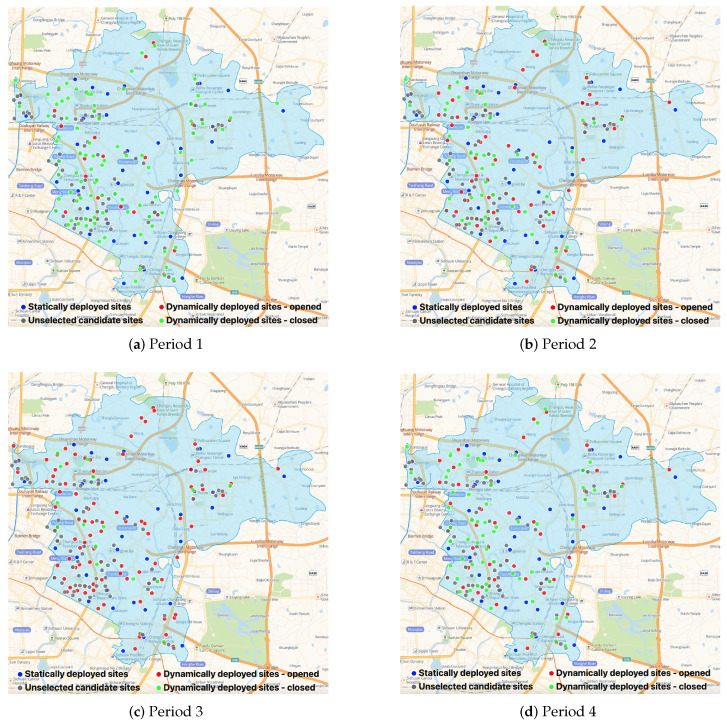
The dynamic site deployment of BS.

**Figure 6:**
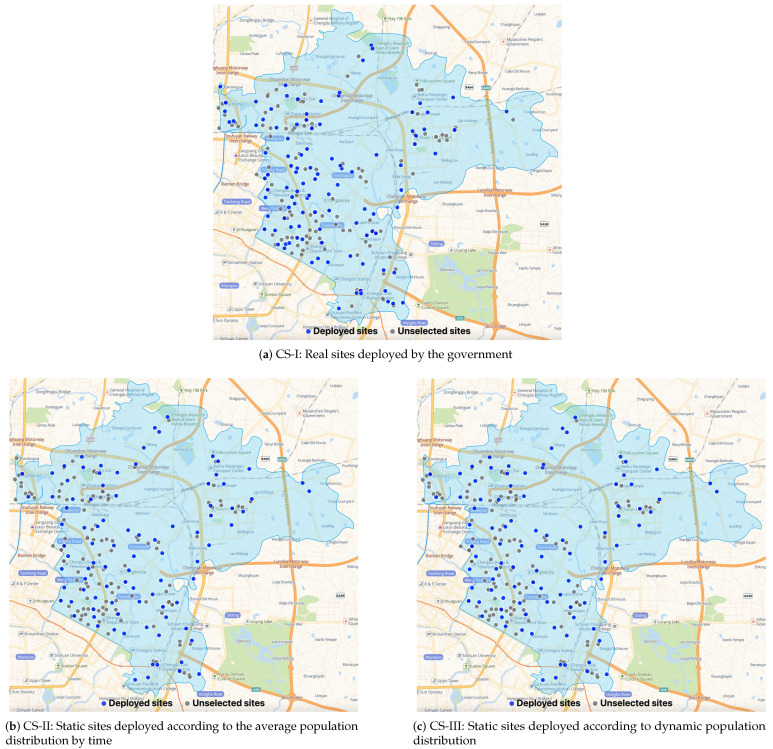
The static site deployment plan of other strategies.

To obtain more insights, we compare the number of various types of NAT sites deployed by various strategies in Table 5 and illustrate the optimal dynamic (static) NAT site deployment plans of BS (CS-I, CS-II, and CS-III) in Figure 5 ( Figure 6). Table 5 shows that BS deploys various numbers of NAT sites in each period, and most NAT sites are deployed at clinics, HCs, and pharmacies. Although the NAT site deployment plans of the three CSs are different, the optimal plans of CS-II and CS-III are nearly the same (CS-III deploys one more Type II site), which explains the various optimal total costs of the four CSs and the similar optimal total costs of CS-II and CS-III. Moreover, from Table 5, we find that reducing the number of Type I sites deployed at hospitals is vital to develop better NAT site deployment plans. This observation is intuitively sound, considering that the fixed cost of Type I NAT sites is relatively high, as shown in Table 2, and that the greater service capacity of Type I sites can lead to serious capacity waste if the testing demands are not high and vary significantly across periods. In addition, since general hospitals normally play an important role in the admission and treatment of ordinary patients and infected patients during epidemics, moving the large-scale testing work out of hospitals can help increase service quality and reduce cross-infection risks in hospitals. Therefore, we suggest avoiding large-scale COVID-19 NAT in general hospitals and mainly using candidate sites with small or medium service capacity for better field practices. Figure 5 and Figure 6 illustrate the spatial distributions of the unselected NAT sites (gray dots, closed in all periods), the statically deployed NAT sites (blue dots, opened in all periods), and the dynamically deployed NAT sites (red dots, opened in that period; green dots, closed in that period). In general, except CS-I, the other strategies deploy NAT sites in the more densely populated areas shown in Figure 4 to ensure that the deployed sites are relatively closer to the demands. The NAT site deployment plan of BS is more responsive to the time-varying demands. For example, in period 1 (i.e., in the morning), the demand is relatively low so the number of opened NAT sites is small to avoid capacity waste. On the contrary, in period 3 (i.e., in the afternoon), the demand becomes relatively high, and more NAT sites are opened to quickly increase the testing capacity. In sum, the comparison between Figure 5 and Figure 6 emphasizes that with a dynamic site deployment strategy, BS has more flexibility to balance testing capacity and demand in each period and to serve the target population better.

### 4.3. Sensitivity Analysis

To examine the impacts of some parameter settings and to obtain more managerial insights, we conduct sensitivity analysis on the factor transferring distance into cost δ and the total number of type *h* staff available in each period $B_{ht}$. Specifically, we conduct the sensitivity analysis via applying various multipliers (the multipliers being 1 indicates our base case setting) on δ and $B_{ht}$ to examine the impacts of δ and $B_{ht}$ on the optimal results of BS (the dynamic site deployment strategy) and CS-III (the best static deployment strategy).

**Figure 7:**
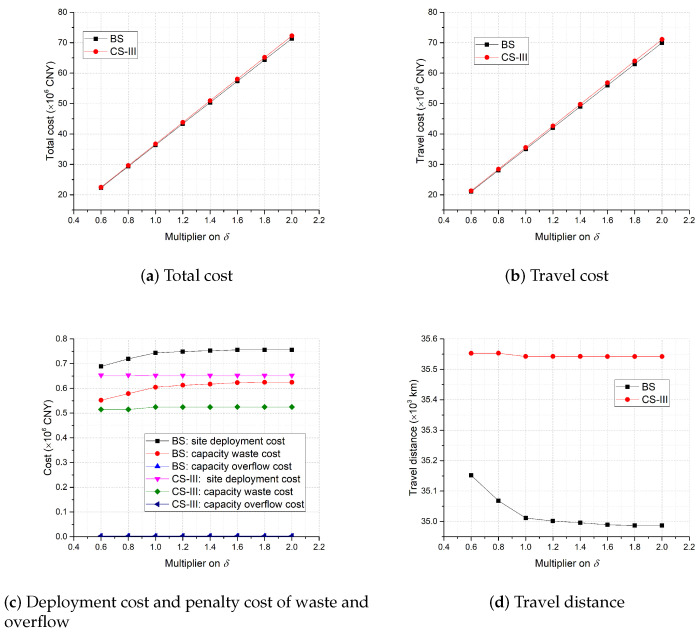
Sensitivity analysis on δ.

The sensitivity analysis results of δ are illustrated in Figure 7. As shown in Figure 7a,b, with the increase of δ (i.e., the unit travel distance cost increases), the total cost and the travel cost of both strategies increase almost linearly, and the total cost and travel cost of BS remain lower than those of CS-III. From Figure 7d, we find that as δ increases, the total travel distance of BS clearly moves down to a limit while that of CS-III reduces slightly, which indicates that the dynamic site deployment strategy can reduce the total travel distance more effectively and make the testing more convenient to the target population. Since the transferring factor δ reflects how a decision-maker emphasizes the importance of site accessibility, Figure 7d suggests that the deployment plan of the dynamic strategy, BS, compared with those of the static strategies, is more sensitive to the decision-maker’s emphasis. Moreover, Figure 7c shows that as δ increases, the site deployment cost and the capacity waste cost of BS rise up to a limit while the other cost components of BS and CS-III are relatively stable. This again highlights that the dynamic site deployment strategy helps trade off the various operational cost components with the travel cost flexibly and subtly to reduce the total cost for better field practices.

**Figure 8:**
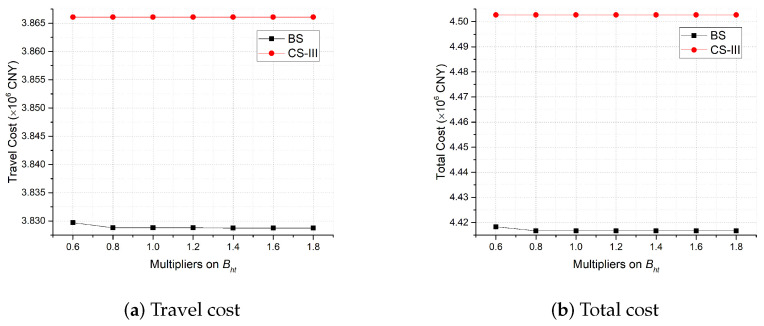
Sensitivity analysis on $B_{ht}$.

Considering that in the practice of large-scale testing, the availability of testing staff usually forms the bottleneck of testing capacity, we also conduct sensitivity analysis on $B_{ht}$, the total number of type *h* staff available in period *t*, to examine its impacts. Figure 8 shows that as $B_{ht}$ increases, the total cost and travel cost of BS move slightly down to a limit, while those of CS-III are nearly unchanged, which not only implicates good robustness of the proposed dynamic strategy when the testing staff are not scarce but also suggests that when $B_{ht}$ is relatively small, preparing more staff for large-scale testing is vital to improving the dynamic site deployment plan; when $B_{ht}$ is relatively large or the static site deployment strategy is implemented, increasing $B_{ht}$ can be marginal for better field practices.

## 5. Conclusions and Future Work

In this study, we propose a multiperiod location-allocation model, which implements a dynamic site deployment strategy, to facilitate the COVID-19 NAT site deployment for COVID-19 control. With a real-world case study, which is based on the Chenghua district of Chengdu, China, we verify the effectiveness and benefits of our proposed model and obtain various managerial insights. For example, deploying NAS sites with the spatial distribution of population has a significant effect on cost reduction, and moving large-scale COVID-19 nucleic acid testing out of general hospitals and utilizing testing sites with small or medium service capacity can contribute to better field practices. Our study emphasizes the importance of deploying NAT sites dynamically for better field practices and reveals that (1) the optimal plan of the dynamic testing site deployment strategy is more flexible and reliable to serve the time-varied testing demands, (2) dynamically deploying NAT sites can help reduce the testing cost and increase the robustness of producing feasible plans with limited medical resources, and (3) decision-makers can obtain various NAT site deployment plans by adjusting the importance of service accessibility.

In the future, several issues related to large-scale COVID-19 NAT can be investigated further. First, a systematic simulation study can be conducted to incorporate more NAT details and justify the real-world applicability of our model. Second, the stochasticity of NAT demand in each period can be further addressed by building a multistage stochastic programming model. Third, various types of testing demands, which can have different infection risks and testing requirements, can be considered to reduce potential cross-infection risks during testing. Fourth, the coordination and cooperation of multiple districts or cities can be investigated to enhance COVID-19 NAT on a greater scale.

## Figures and Tables

**Table 1:** Notations.

Sets
T={1,2,...,τ}, periods, indexed by *t*
*I*, COVID-19 NAT demand points, indexed by *i*
*J*, candidate COVID-19 NAT sites, indexed by *j*
*K*, types of medical supplies for COVID-19 NAT, indexed by *k*
*H*, types of staff for COVID-19 NAT, indexed by *h*
**Parameters**
$D_{it}$, demand amount, number of target people needing COVID-19 NAT, at demand point *i* in period *t*
$l_{ij}$, distance between demand point *i* and candidate NAT site *j*
$a_{kj}$, amount of types *k* medical supplies required to run candidate NAT site *j* per period
$b_{hj}$, number of type *h* staff required to run candidate NAT site *j* per period
$C_{j}$, the maximum amount of demand that can be served per period at candidate NAT site *j*
$f_{j}$, fixed cost of deploying NAT site *j*
$A_{kt}$, total amount of types *k* medical supplies available in period *t*
$B_{ht}$, total number of type *h* staff available in period *t*
$\alpha_{j}$, unit penalty cost of wasting NAT capacity of opened candidate NAT site *j*
$\beta_{j}$, unit penalty cost of NAT capacity overflow of opened candidate NAT site *j*
δ, a factor transferring distance into cost
*M*, a huge positive number
**Decision Variables**
$y_{jt}\in \left\{0,1\right\}$, equals 1 if candidate NAT site *j* opens in period *t*; 0, otherwise
$x_{ijt}\geq 0$, number of target people of demand point *i* moving to candidate NAT site *j* in period *t*
$c_{jt}^{-}\geq 0$, amount of capacity left at opened candidate NAT site *j* in period *t*
$c_{jt}^{+}\geq 0$, amount of capacity overflow at opened candidate NAT site *j* in period *t*

**Table 2:** Parameter settings for the various types of candidate NAT sites.

Type	Capacity $C_{j}$	Cost $f_{j}$	$\alpha_{j}$	$\beta_{j}$	$a_{\mathrm{kj}}$		$b_{\mathrm{hj}}$	
					k=1	k=2	h=1	h=2
I	2000	2000	1	4	10	800	3	5
II	800	1400	1	4	8	500	2	3
III	400	800	3	2	3	300	1	1
IV	400	800	3	2	4	300	1	2
V	400	1000	5	2	5	300	1	3

**Table 3:** Parameter settings of $A_{kt}$ and $B_{ht}$.

Period	$A_{\mathrm{kt}}$		$B_{\mathrm{ht}}$	
	k=1	k=2	h=1	h=2
1	2000	60,000	400	600
2	2000	80,000	300	800
3	2000	80,000	300	800
4	2000	60,000	400	600

**Table 4:** Optimal results of various site deployment strategies.

Strategies	Cost Components ($\times 10^{6}$ CNY)				Total Cost ($\times 10^{6}$ CNY)
	Deployment	Capacity Waste	Capacity Overflow	Travel	
BS	0.364	0.221	0.003	3.829	4.417
CS-I	-	-	-	-	(infeasible)
CS-I (3.5 km)	0.557	0.448	0.011	4.182	5.198
CS-II	0.380	0.242	0.008	3.874	4.504
CS-III	0.386	0.245	0.006	3.866	4.503

**Table 5:** The number of various types of sites deployed by various strategies.

Strategies	Periods	Hospitals (57)	Clinics (68)	HCs (41)	Pharmacies (44)	Other Sites (22)
BS	$T_{1}$	0	13	17	33	1
	$T_{2}$	3	28	26	29	8
	$T_{3}$	10	37	31	34	14
	$T_{4}$	2	20	21	30	5
CS-I (3.5 km)	-	25	28	17	18	22
CS-II	-	3	27	24	30	8
CS-III	-	3	28	24	30	8

## Data Availability

Data will be made available on request.

## Abbreviations

The following abbreviations are used in this manuscript:

NAT	Nucleic acid testing
EHF	Emergency healthcare facility
BS	Base strategy
CS	Comparison strategy
